# A Case of Superfœtation

**Published:** 1880-07

**Authors:** D. A. Walden

**Affiliations:** Beatrice, Neb.


### ARTICLE VI.

*A Case of Superfœtation.*

Mrs. G. aged 20, primapara, married about ten months, was taken with slight labor pains on the evening of April 18th.

I was called at 3 o'clock p. m. on the 19th; after a short but severe labor she was delivered of a healthy, full-termed female child of about five pounds weight. The placenta was delivered without any unusual difficulty. I called on the following morning and found the patient in good spirits and free from pain.

About 9 o'clock p. m. on the 21st, while using the vessel, there passed from her a complete, unruptured sac containing a male fœtus, and placenta, in appearance of about four months gestation. (We have the fœtus in our possession.) The following afternoon saw her in an easy and painless condition.

She has made a rapid recovery, and the child is healthy. Upon questioning her she stated that she had her menstrual flow for three or four months after she was aware of her pregnancy. A close inspection revealed no evidence of a double uterus.

Playfair in his "System of Midwifery" page 162, quotes from Tyler Smith as follows:

"A young married woman, pregnant for the first time, miscarried at the end of the fifth month, and some hours afterward, a small clot was discovered, inclosing a perfectly healthy ovum of about one month. There were no signs of a double uterus in this case. The patient had menstruated regularly during the time she had been pregnant." To this the author adds:

"This case is of special interest from the fact of the patient having menstruated during pregnancy—a circumstance only explicable on the same anatomical grounds which render superfœtation possible. So far as I know, it is the only instance in which the concidence of superfœtation and menstruation during early pregnancy has been observed."

We find that eminent authors, as Churchill, Ramsbotham and Hunter, claim that superfœtation is impossible.

We are inclined to the belief that Playfair's explanation on pages 162–163 bears us out on the opinion that this is a true case of superfœtation.

D. A. WALDEN, M.D.

BEATRICE, Neb., June 18, 1880.

---

THE active state of the constrictor cervicis muscle is in the period of gestation, during which process it is transformed into the constrictor oris uteri, but in the dormant condition of the unimpregnated uterus this muscle becomes very often like an idle individual, mischievous, converting by morbid compression the veins underlying it, not into mythical, but into actual closed Pandora boxes. From the very beginning to the end of pregnancy, the life of this muscle is evolution and growth, and its office that of a support, whereby it is prepared for a supreme effort during and after parturition ; but in the dormant state of the unimpregnated uterus its slow, feeble, and stealthy, but persistent, contractions are, when they occur, always morbid. The relation this constrictor muscle bears to the veins of the cervix, and the agency or *vis morbi* it possesses of becoming by closing them a *causa morborum*, thereby producing multiform diseases, have never, so far as is known, been even hinted at, much less described, by any medical, surgical, or physio-pathological writer. —*T. H. Buckley in his Notes on the Anatomical Relations of the Uterine Structure.*

